# Impairment of insulin-stimulated Akt/GLUT4 signaling is associated with cardiac contractile dysfunction and aggravates I/R injury in STZ-diabetic Rats

**DOI:** 10.1186/1423-0127-16-77

**Published:** 2009-08-25

**Authors:** Jiung-Pang Huang, Shiang-Suo Huang, Jen-Ying Deng, Li-Man Hung

**Affiliations:** 1 Department of Life Science, College of Medicine, Chang Gung University, Tao-Yuan, Taiwan, Republic of China; 2 Department of Pharmacology and Institute of Medicine, College of Medicine, Chung Shan Medical University, Taichung, Taiwan, Republic of China

## Abstract

In this study, we established systemic in-vivo evidence from molecular to organism level to explain how diabetes can aggravate myocardial ischemia-reperfusion (I/R) injury and revealed the role of insulin signaling (with specific focus on Akt/GLUT4 signaling molecules). The myocardial I/R injury was induced by the left main coronary artery occlusion for 1 hr and then 3 hr reperfusion in control, streptozotocin (STZ)-induced insulinopenic diabetes, and insulin-treated diabetic rats. The diabetic rats showed a significant decrease in heart rate, and a prolonged isovolumic relaxation (tau) which lead to decrease in cardiac output (CO) without changing total peripheral resistance (TPR). The phosphorylated Akt and glucose transporter 4 (GLUT 4) protein levels were dramatically reduced in both I/R and non-I/R diabetic rat hearts. Insulin treatment in diabetes showed improvement of contractile function as well as partially increased Akt phosphorylation and GLUT 4 protein levels. In the animals subjected to I/R, the mortality rates were 25%, 65%, and 33% in the control, diabetic, and insulin-treated diabetic group respectively. The I/R-induced arrhythmias and myocardial infarction did not differ significantly between the control and the diabetic groups. Consistent with its anti-hyperglycemic effects, insulin significantly reduced I/R-induced arrhythmias but had no effect on I/R-induced infarctions. Diabetic rat with I/R exhibited the worse hemodynamic outcome, which included systolic and diastolic dysfunctions. Insulin treatment only partially improved diastolic functions and elevated P-Akt and GLUT 4 protein levels. Our results indicate that cardiac contractile dysfunction caused by a defect in insulin-stimulated Akt/GLUT4 may be a major reason for the high mortality rate in I/R injured diabetic rats.

## Background

Diabetes mellitus is the world's fastest-growing disease with high morbidity and mortality rates predominantly as results of cardiovascular diseases [1,2]. Prospective studies have documented increased likelihood of sudden cardiac death and unrecognized myocardial infarctions in patients with diabetes [3]. Moreover, acute ischemic syndromes, peripheral arterial disease, and advanced cardiovascular disease (CVD) complications occur more commonly in patients with diabetes than in those without [3]. Despite the prevalence and the significant impact this disease has on our world today, the understanding of the cellular and molecular perturbations that predispose to altered myocardial structure and function remains incomplete. Recently, it has been speculated that myocardial insulin resistance develops in animal models of both type 1 and type 2 diabetes [4]. Various studies to date indicate multiple sites of impaired insulin signaling in various animal models; all the findings clearly support the existence of myocardial insulin resistance [4]. Insulin-stimulated glucose uptake, protein synthesis and glycogen synthesis have been shown to be reduced in the heart and cardiomyocytes of diabetic rats [5,6].

Insulin regulates metabolism in the heart by modulating glucose transport, glycolysis, glycogen synthesis, lipid metabolism, protein synthesis, growth, contractility, and apoptosis in cardiomyocytes [7-9]. In addition, vasodilator actions of insulin in coronary vasculature augment myocardial perfusion [10]. As in other insulin-sensitive tissues, insulin signaling via PI3K/Akt pathways plays a key role in cardiac glucose uptake [7]. Insulin resistance is an important risk factor for the development of hypertension, atherosclerotic heart disease, left ventricular hypertrophy and dysfunction, and heart failure [11]. It reflects a disturbance of glucose metabolism and can potentially worsen metabolic efficiency of cardiomyocytes. During insulin resistance or diabetes, the heart rapidly modifies its energy metabolism to decrease glucose utilization and increase fatty acid consumption. Accumulating evidence suggests that this alteration of cardiac metabolism plays an important role in the development of cardiomyopathy [11]. Heart disease is the leading cause of death in diabetic patients [12], with coronary artery disease (CAD) and atherosclerosis being the primary reasons for increased incidence of cardiovascular dysfunction [12,13]. A predisposition to heart failure might also reflect the effects of underlying abnormalities in cardiac diastolic function that can be detected in asymptomatic patients with diabetes [14,15]. Several etiological factors have been put forward to explain why hyperglycemia and/or diabetes tend to lead to diabetic cardiomyopathy. However, the underlying mechanisms are not yet fully understood. Therefore, this study aims to investigate the mechanisms of diabetes which causes cardiac dysfunctions in STZ-diabetic rats with and without myocardial I/R injuries. Our study yielded systemic in-vivo evidence in different structural levels to explain how diabetes aggravate myocardial I/R injury and reveal the role of insulin signaling.

## Materials and methods

### Experimental Diabetic Animals

This investigation abides by the rules written in the Guide for the Care and Use of Laboratory Animals, published by the US National Institutes of Health (NIH publication No. 85-23, revised 1996). Eight week-old Sprague-Dawley (SD) rats (body weight from 250 gm to 300 gm) were maintained in the Animal Center of the Chang Gung University, under an ambient temperature of 25 ± 1°C and a light-dark period of 12 hrs. The animals were fed with normal chow and water.

To induce diabetes, male SD rats were fasted and anesthetized by intraperitoneal injection of pentobarbital with a dosage of 65 mg/kg. The animals were then injected with a single intravenous injection of freshly prepared streptozotocin (STZ, 65 mg/kg). STZ has been shown to destroy pancreatic β-cells and induce a diabetic state during the first 24 h after injection [16]. Two weeks later, blood sugar levels were measured by the glucose oxidase method (Chemistry Analyzer; Auto analyzer Quik-Lab., Ames, Spain). Animals with blood sugar levels above 300 mg/dl and symptoms of polyphagia, polyuria and polydipsia were classified as being induced to diabetes. Age-matched rats were used as nondiabetic controls.

### Myocardial I/R Injury

SD rats were anesthetized with inactin (100 mg/kg i.p.) and urethane (400 mg/kg i.p.). Myocardial ischemia was performed by a temporary tightening of the silk ligature around the left main coronary artery as previously described [17,18]. Reperfusion was achieved by releasing the tension applying to the ligature (operated groups). Sham operated animals underwent all the same surgical procedures, except for the silk that passes around the left coronary artery being not tied (sham groups). Before and during the ischemia and reperfusion period, heart rate (HR), blood pressure (BP) and ECG changes were recorded. Ventricular ectopic activity was evaluated according to the diagnostic criteria advocated on the Lambeth Convention [19]. The incidence and duration of ventricular tachyarrhythmias, including ventricular tachycardia (VT) and ventricular fibrillation (VF), in the surviving animals were documented.

### Myocardial Infarction

Areas of infarction and perfusion were determined by the triphenyl tetrazolium chloride-Evan's blue technique [20]. At the end of the experiments, the coronary artery was re-occluded and 2.0 ml methyl blue (3%) was injected intravenously to mark the area at risk. The heart was then excised and the atria were removed. The ventricular tissue was sliced into 1 mm sections and incubated in tetrazolium dye (1% 2, 3, 5-triphenyltetrazolium chloride/0.9% NaCl, pH 7.4) at 37°C for 40 minutes. Sections were then placed in 10% formaldehyde in saline for 2 days before infarct (white) tissue was excised. The infarct tissue was quantified as percentage of occluded zone.

### Hemodynamic Measurements

The animals were anesthetized with inactin (100 mg/kg i.p.) and urethane (400 mg/kg i.p.) then placed on controlled heating pads (TC-1000 Temperature Controller, CWE Inc. USA) with the core temperature measured via a rectal probe maintained at 37°C. A microtip pressure-volume catheter (SPR-838; Millar Instruments, Houston, TX) was inserted into the right common carotid artery and advanced into the left ventricle (LV) under pressure control as described [21-23]. Polyethylene cannulas (PE-50) were inserted into the right femoral artery for the measurement of mean arterial pressure (MAP). After stabilizing for 20 min, the signals were continuously recorded at a sampling rate of 1,000/s by using an ARIA pressure-volume (P-V) conductance system (Millar Instruments) coupled to a Powerlab/4SP analog-to-digital converter (AD Instruments, Mountain View, CA). Data was displayed and recorded on a computer. All P-V loop data were analyzed by using a cardiac P-V analysis program (PVAN3.2; Millar Instruments), and the heart rate (HR), end-systolic volume (ESV), end-diastolic volume (EDV), end-systolic pressure (ESP), end-diastolic pressure (EDP), stroke volume (SV), ejection fraction (EF), cardiac output (CO), stroke work (SW), arterial elastance (Ea; end-systolic pressure/SV), mean arterial pressure (MAP), maximal slope of systolic pressure increment (max dP/dt), and diastolic decrement (min dP/dt) were computed. The relaxation time constant (tau), an index of diastolic function, was calculated by two different methods [Weiss method: regression of log (pressure) versus time and Glantz method: regression of dP/dt vs. pressure] using PVAN3.2. Total peripheral resistance (TPR) was calculated by the following equation: TPR = MAP/CO. The hemodynamic parameters were also determined under conditions of changing preload, elicited by transiently compressing the inferior vena cava (IVC) using a cotton swab inserted through a small, transverse, upper abdominal incision. This technique yields reproducible occlusions in animals without opening the chest cavity. Because max dP/dt may be preload dependent, the P-V loops recorded at different preloads were used to derive other useful systolic function indexes that may be less influenced by loading conditions and cardiac mass. These measurements include dP/dt-end diastolic volume (EDV) relation (dP/dt-EDV), end-systolic PV relation [maximum chamber elasticity (ESPVR), *E*max], and the preload-recruitable stroke work (PRSW), which represents the slope between SW and EDV and is independent of chamber size and mass. The slope of the end-diastolic PV relationship (EDPVR), an index of LV stiffness, was also calculated from P-V relations using PVAN 3.2.

### Immunoblotting

Tissue lysates (membranous and cytoplasmic fraction) were isolated from cardiac tissues in accordance to previously published procedure with slight modifications [24]. In brief, tissues were first homogenized in a lysis buffer (M-PER; Pierce, USA) with 1 mM phenylmethylsulfonylfluoride (PMSF) as a protease inhibitor. The tissue lysates were then ultracentrifuged at 50,000 rpm for 1 h at 4°C. The resulting supernatant was labeled as a cytoplasmic fraction. The resulting pellet, which contained the crude membrane, was re-suspended in M-PER (300~500 μl) with 0.5% Triton X-100, incubated at 4°C overnight, and centrifuged again at 15,700 g for 20 min. Finally, the supernatant was collected and labeled as a membrane fraction. Protein samples of cytoplasmic and membranous lysates were subjected to 10% SDS-PAGE and electrophoretically transferred to PVDF protein sequencing membrane for 2 hrs. The membrane was blocked in 5% non-fat milk in Tris-buffered saline with 0.1% Tween-20 (TBST). It was then washed and blotted with anti-GLUT1 (Chemicon, USA) and GLUT4 (Chemicon, USA). Phosphorylation of Akt was detected with anti-phospho-Akt (Ser473) and anti-phospho-Akt (Thr308) (Santa Cruz); Akt was determined with anti-Akt antibody (Santa Cruz). The membrane was then incubated with HRP-conjugated secondary antibody prior to chemiluminescence detection (Pierce, USA).

### Statistical Analysis

The difference in BP and HR, duration of VT and VF, infarct size, GLUTs, and Akt protein levels between control, diabetic, and insulin treated diabetic groups were analyzed by Student's *t*-test. The difference in the mortality rate was analyzed with a Chi-square test. *P *< 0.05 was used as criteria for statistical significance.

## Results

### The Influences of Hyperglycemia on Cardiovascular System

Male SD rats were intravenously injected with streptozotocin (STZ) to induce insulinopenic diabetes which was characterized by elevations in plasma glucose level above 300 mg/dl and by exhibiting symptoms of polyphagia, polyuria, and polydipsia. Animals treated with STZ resulted in consistent hyperglycemia and hypoinsulinemia that persisted over the 3 week period. Plasma glucose level in diabetic rats was 606 ± 16 mg/dl compared with values of 169 ± 4 mg/dl in age-matched controls, *P *< 0.001. As expected, the diabetic rats had lower body weights and ventricular weights compared to age-matched controls. Body weight loss and hyperglycemia induced by diabetes were markedly restored by insulin treatment of 5 days (Table 1). Diabetic rats also showed a lower MAP and a higher heart to body weight ratio than age-matched controls. Insulin treatment not only affected metabolic parameters (including reduced plasma glucose level and attenuated body weight loss) but also significantly elevated MAP as shown in the comparison between the two groups (Table 1).

**Table 1:** Laboratory characteristics in age-matched control, diabetic (DM), and insulin-treated diabetic (DMI) rats.

	Control (n = 21)	DM (n = 36)	DMI (n = 25)
Plasma glucose (mg/dl)	169 ± 4	607 ± 16*^(P < 0.001)^	397 ± 24^†(P < 0.001)^
Plasma insulin (μg/L)	2.7 ± 0.22	1.08 ± 0.16*^(P < 0.001)^	3.29 ± 0.27^†(P < 0.001)^
Body weight (g)	411 ± 13	235 ± 5*^(P < 0.001)^	252 ± 5^†(P < 0.05)^
Heart weight (g)	1.32 ± 0.05	0.89 ± 0.03*^(P < 0.001)^	0.97 ± 0.03^†(P < 0.05)^
Ratio heart/body weight (mg/g)	3.51 ± 0.008	3.91 ± 0.012*^(P < 0.001)^	3.85 ± 0.013
MAP (mmHg)	118 ± 5	94 ± 4*^(P < 0.001)^	108 ± 5^†(P < 0.05)^
TPR (mmHg/ml/min)	2.21 ± 0.1	2.42 ± 0.09	2.21 ± 0.1

Data are expressed as means ± SEM (*, vs. control; †, vs. DM). DM, STZ-diabetes; DMI, insulin-treated diabetes; MAP, mean arterial pressure; TPR, total peripheral resistance.

Hyperglycemia and hypoinsulinemia may have divergent or distinct effects on the progression of cardiomyopathy in STZ-diabetic rats. We sought to directly measure the cardiac performance by Millar pressure-volume instruments. The effects of diabetes and insulin treatment on basic hemodynamic variables are shown in Table 2. Induction of insulin-deficient diabetes for 3 weeks decreased basal heart rate (HR) by 16.3%, cardiac output (CO) by 27.3% and prolonged relaxation time constant (tau, an index of diastolic function) by 17.4% under basal condition. The slope of ESPVR (end-systolic elastance, Ees; indicator of left ventricular contractility) was also decreased by 28.5% in diabetic rats. The maximum pressure (peak LV pressure), end diastolic pressure (EDP) and estimated peak isovolumic pressure (P@dPdt max) was not altered. The diabetes-induced slower HR and lower CO were reversed by 1 week of insulin treatment. Additionally, insulin treated diabetic rats showed an increase in EDP (36.2%) and peak LV pressure (11.4%), and a rise of 9.9% in estimated peak isovolumic pressure (Table 2).

**Table 2:** Baseline hemodynamic parameters in age-matched control, diabetic (DM), and insulin-treated diabetic (DMI) rats measured by Millar pressure-volume conductance catheter system.

	Control (n = 24)	DM (n = 28)	DMI (n = 21)
Heart rate (bpm)	392 ± 7	328 ± 8*^(P < 0.01)^	352 ± 8^†(P < 0.05)^
End-systolic volume (μL)	194 ± 16	183 ± 16	185 ± 14
End-diastolic volume (μL)	292 ± 19	256 ± 14	254 ± 15
Maximum pressure (mmHg)	110 ± 3.8	114 ± 3.8	127 ± 5.1^†(P < 0.05)^
Minimum pressure (mmHg)	2.49 ± 0.62	1.87 ± 0.26	3.22 ± 0.36^†(P < 0.01)^
End-systolic pressure (mmHg)	101 ± 3.9	109 ± 4.2	122 ± 5.4
End-diastolic pressure (mmHg)	5.66 ± 0.56	5 ± 0.32	6.81 ± 0.5^†(P < 0.01)^
Stroke volume (μL)	136 ± 12.5	118 ± 7.1	139 ± 7.8
Ejection fraction (%)	43.7 ± 3.14	44.5 ± 2.87	48.4 ± 2.38
Cardiac output (μL/min)	53,414 ± 5,133	38,810 ± 2,712*^(P < 0.05)^	48,765 ± 2,942^†(P < 0.05)^
Stroke work (mmHg*μL)	11,088 ± 1,837	9,073 ± 785	11,025 ± 824
Arterial elastance (mmHg/μL)	0.93 ± 0.12	1.03 ± 0.08	0.95 ± 0.07
End-systolic elastance (mmHg/μL)	0.94 ± 0.09	0.67 ± 0.07*^(P < 0.05)^	0.63 ± 0.05
dPdt max (mmHg/sec)	7,534 ± 460	7,773 ± 389	8,899 ± 388
dPdt min (mmHg/sec)	-8,192 ± 600	-7,144 ± 376	-7,960 ± 413
P@dPdt max (mmHg)	74 ± 3.7	75 ± 2.3	82 ± 2.7^†(P < 0.05)^
Tau_w (msec)	10.33 ± 0.35	12.13 ± 0.33*^(P < 0.01)^	11.344 ± 0.28
Tau_g (msec)	11.9 ± 0.36	14.76 ± 0.55*^(P < 0.01)^	14.68 ± 0.7

Data are expressed as means ± SEM (*, vs. control; †, vs. DM). DM, STZ-diabetes; DMI, insulin-treated diabetes.

### The Effects of Diabetes on I/R-induced Mortality Rate and Rhythm Disturbances

In the control (n = 18) and diabetic (n = 37) groups, the I/R-induced mortality rates were 25% and 64.5% respectively (*P *< 0.05). Consistent with antihyperglycemic effect, insulin significantly reduced I/R-induced mortality rate from 64.5% to 33.3% (*P *< 0.05, n = 15).

**Figure 1:**
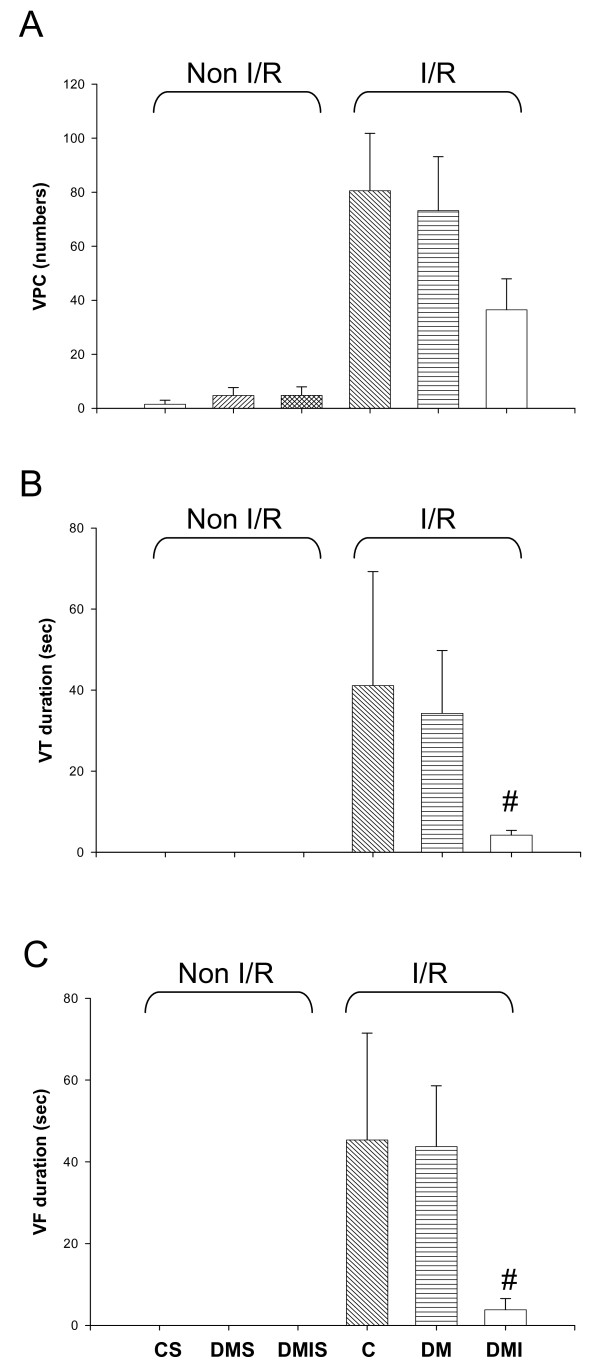
**The effects of diabetes on I/R-induced ventricular rhythm disturbances including VPC (A), VT (B), and VF (C) were determined during ischemic period (1 h) in the age-matched controlled (n = 7), STZ-diabetic (n = 28), and insulin-treated diabetic (n = 9) rats**. Data were expressed as means ± SEM, # *P *< 0.05 vs. DM. C, age-matched control; CS, control sham; DM, STZ-induced diabetes; DMS, DM sham; DMI, diabetes treated with insulin (4 IU/day for 5 days); DMIS, DMI sham; I/R, myocardial ischemia 1 h following by a 3 h of reperfusion; VPC, ventricular premature complexes; VT, ventricular tachycardia; VF, ventricular fibrillation.

In the control group, severe ventricular arrhythmias began 6-7 minutes after coronary artery occlusion, peaked around 8-12 minutes, and subsided at approximately 15 minutes. The duration of VF was calculated to be 45.35 ± 26.15 seconds, and the duration of VT was 41.1 ± 28.17 seconds (Figure 1). During the 1 h ischemia period, the numbers of ventricular premature complexes (VPC) was 80.57 ± 21.25. Amongst the 7 rats in the control group, 3 animals (42.86%) exhibited VF, and 6 animals (85.71%) exhibited VT (Figure 1). There was no significant difference between the severities of the arrhythmias between the groups. However, diabetic rats treated with insulin resulted in a decrease of the duration of VT and VF from 34.3 ± 15.6 and 43.7 ± 14.9 to 4.22 ± 1.17 and 3.83 ± 2.75 seconds respectively (Figure 1).

### The Effects of Diabetes on I/R-induced Myocardial Infarction

**Figure 2:**
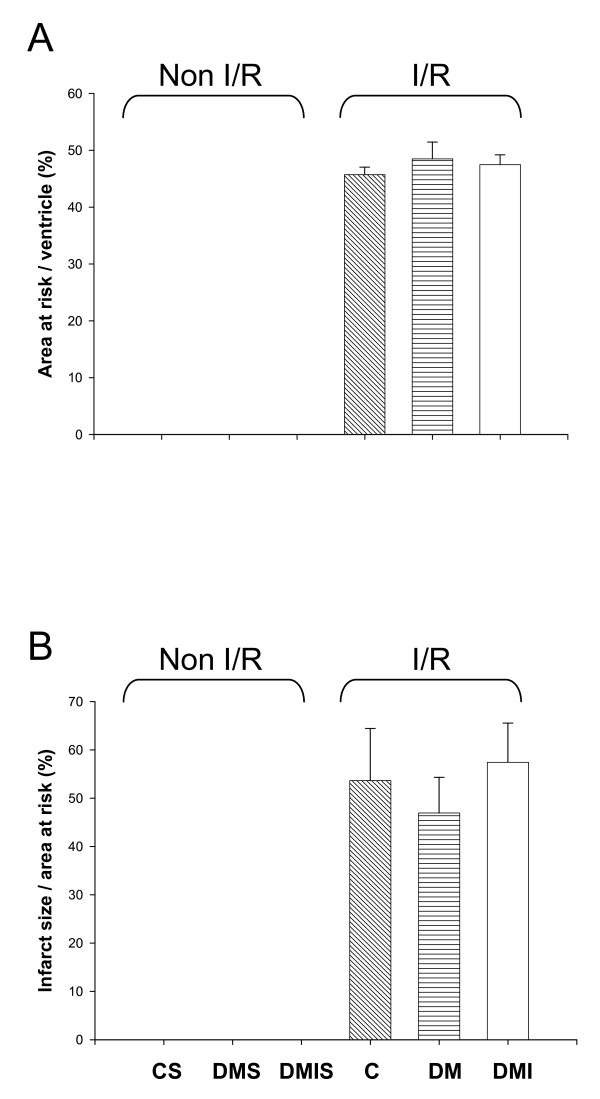
**The effects of diabetes on I/R-induced myocardial infarction were evaluated in the age-matched controlled (n = 7), STZ-diabetic (n = 10), and insulin-treated diabetic (n = 9) rats**. SD rats were subjected to 1 h of left coronary artery occlusion and 3 h of reperfusion. The level of myocardial infarction was determined by the triphenyl tetrazolium chloride-Evan's blue technique. (A) Ratio of area at risk to total ventricle. (B) Ratio of infarct size to area at risk. Data were expressed as mean ± SE. C, age-matched control; CS, control sham; DM, STZ-induced diabetes; DMS, DM sham; DMI, diabetes treated with insulin (4 IU/day for 5 days); DMIS, DMI sham; I/R, myocardial ischemia 1 h following by a 3 h of reperfusion.

The ischemic area and infarct size were estimated after 60 minutes of ischemia and 3 hours of reperfusion. Between the experimental groups, there were no significant differences in the size of area at risk (Figure 2A), indicating that similar amount of tissue was jeopardized by the occlusion of the left coronary artery in each group. After I/R, the necrotic area make up 53.65 ± 10.79% and 46.93 ± 7.39% of the area at risk in the control and diabetic groups respectively. Insulin treatment did not yield any improving effects on I/R-induced infarct size when compared to the diabetic group (Figure 2B).

### The Effects of Diabetes on I/R-induced Hemodynamic Changes

The effects of insulin-deficient diabetes on I/R-induced hemodynamic variables are shown in Table 3. In the control group, animals subjected to I/R had a reduction of peak LV pressure, ejection fraction, CO, maximal slope of systolic pressure increment (dP/dt max), diastolic decrement (dP/dt min), and estimated peak isovolumic pressure (Table 2 and 3). In the STZ-diabetic group, I/R-decreased cardiac contractility was exacerbated more in diabetic rats than in non-diabetic controls (Table 3); these effects were partially antagonized by 5 days Insulin treatment. Insulin-treated diabetic rats exhibited a significant rise of 69.8% in Ees (load-independent index of contractility) and a shortening of 39.9% in relaxation time constant (tau_g) compared with diabetic rats (Table 3).

**Table 3:** I/R-induced hemodynamic changes in age-matched control, diabetic (DM), and insulin-treated diabetic (DMI) rats measured by Millar pressure-volume conductance catheter system.

	Control (n = 7)	DM (n = 9)	DMI (n = 8)
Heart rate (bpm)	366 ± 12	263 ± 12*^(P < 0.001)^	299 ± 12
End-systolic volume (μL)	253 ± 42	233 ± 35	219 ± 37
End-diastolic volume (μL)	311 ± 45	286 ± 32	279 ± 39
Maximum pressure (mmHg)	90 ± 3	71 ± 4.4*^(P < 0.01)^	75 ± 4.4
Minimum pressure (mmHg)	2.54 ± 0.46	1.74 ± 0.4	2.86 ± 1.86
End-systolic pressure (mmHg)	87 ± 2.9	66 ± 4.9*^(P < 0.01)^	70 ± 3.9
End-diastolic pressure (mmHg)	6.98 ± 0.92	4.9 ± 0.41*^(P < 0.05)^	6.76 ± 2.02
Stroke volume (μL)	94 ± 13	84 ± 9.2	92 ± 9.3
Ejection fraction (%)	30.8 ± 4.53	31.3 ± 4.44	33.4 ± 3.95
Cardiac output (μL/min)	33,914 ± 4,240	22,504 ± 3,263*^(P < 0.05)^	27,342 ± 2,848
Stroke work (mmHg*μL)	5,501 ± 914	4,031 ± 601	4,640 ± 720
Arterial elastance (mmHg/μL)	1.04 ± 0.15	0.9 ± 0.17	0.82 ± 0.07
End-systolic elastance (mmHg/μL)	0.893 ± 0.179	0.484 ± 0.071*^(P < 0.05)^	0.822 ± 0.166
dPdt max (mmHg/sec)	4,956 ± 292	3,738 ± 298*^(P < 0.05)^	4,523 ± 639
dPdt min (mmHg/sec)	-3,944 ± 279	-3,002 ± 414	-3,054 ± 303
P@dPdt max (mmHg)	49.53 ± 3.25	39.19 ± 2.93	42.91 ± 4.54
Tau_w (msec)	11.44 ± 0.86	16.71 ± 1.29*^(P < 0.1)^	13.51 ± 1.45
Tau_g (msec)	14.73 ± 1.65	24.07 ± 0.92*^(P < 0.05)^	16.62 ± 0.71^†(P < 0.05)^

Data are expressed as means ± SEM (*, vs. control; †, vs. DM). DM, STZ-diabetes; DMI, insulin-treated diabetes.

### The Effects of Diabetes and I/R injury on Glucose Transporters (GLUTs) Membrane Translocation and Akt Phosphorylation

**Figure 3:**
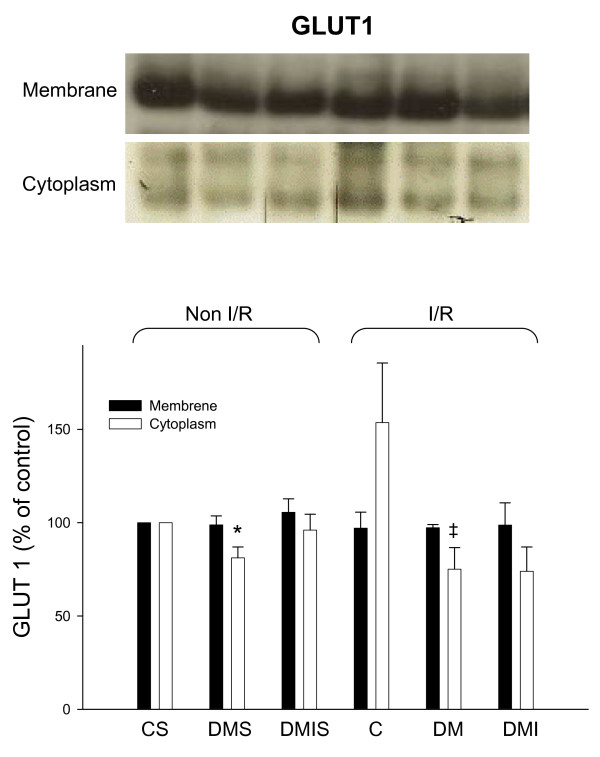
**The influences of diabetes on cytoplasmic and membranous GLUT1 protein level were examined in the age-matched controlled, STZ-diabetic, and insulin-treated diabetic rats with or without I/R injury**. Equal amounts of proteins were resolved on 10% SDS-PAGE and blotted with respective GLUT1 antibodies. All experiments were performed in triplicates from six animals (*, vs. CS; ‡, vs. C; n = 6 per group). The upper panels show blots and the lower panels show quantified ratio. C) age-matched control; CS, control sham; DM, STZ-induced diabetes; DMS, DM sham; DMI, diabetes treated with insulin (4 IU/day for 5 days); DMIS, DMI sham; I/R, myocardial ischemia 1 h following by a 3 h of reperfusion.

**Figure 4:**
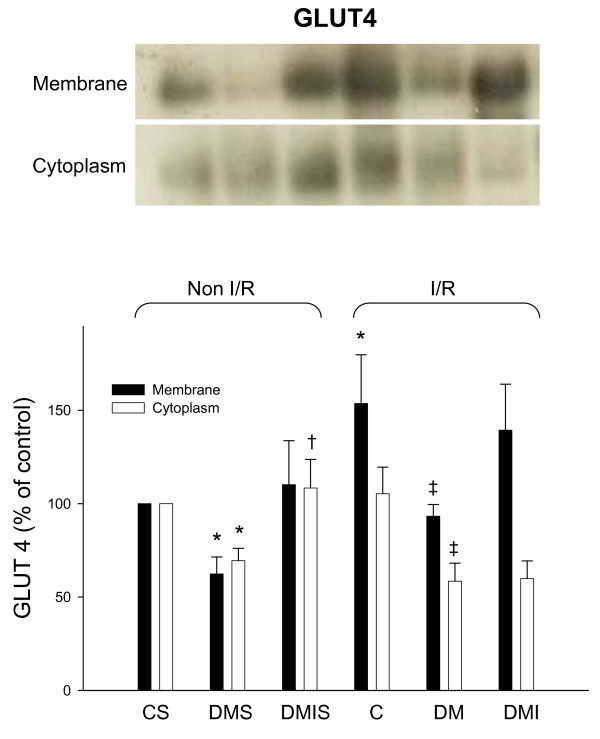
**The influences of diabetes on cytoplasmic and membranous GLUT4 protein level were examined in the age-matched controlled, STZ-diabetic, and insulin-treated diabetic rats with or without I/R injury**. Equal amounts of proteins were resolved on 10% SDS-PAGE and blotted with respective GLUT4 antibodies. All experiments were performed in triplicates from six animals (*, vs. CS; †, vs. DMS; ‡, vs. C; n = 6 per group). Upper panels show blots, and quantified ratio is shown in the lower panels. C, age-matched control; CS, control sham; DM, STZ-induced diabetes; DMS, DM sham; DMI, diabetes treated with insulin (4 IU/day for 5 days); DMIS, DMI sham; I/R, myocardial ischemia 1 h following by a 3 h of reperfusion.

The membranous and cytoplasmic GLUTs protein levels were examined by immunoblotting method in experimental rat heart with and without I/R injuries respectively. Diabetic rats had a dramatic reduction of cytoplasmic GLUT1 protein levels in both I/R and non-I/R rat hearts compared to the controls (Figure 3). In contrast, the membranous GLUT1 protein levels were not significantly different between the groups. The cytoplasmic and membranous GLUT4 protein levels were significantly reduced in both I/R and non-I/R diabetic rat hearts. Insulin only partially reversed the diabetic effects on GLUT4 protein expression in STZ-diabetic rat hearts (Figure 4). Notably, with the exception of the insulin-treated group, I/R also dramatically elevated membranous GLUT4 protein levels compared to the sham-control group (Figure 4).

**Figure 5:**
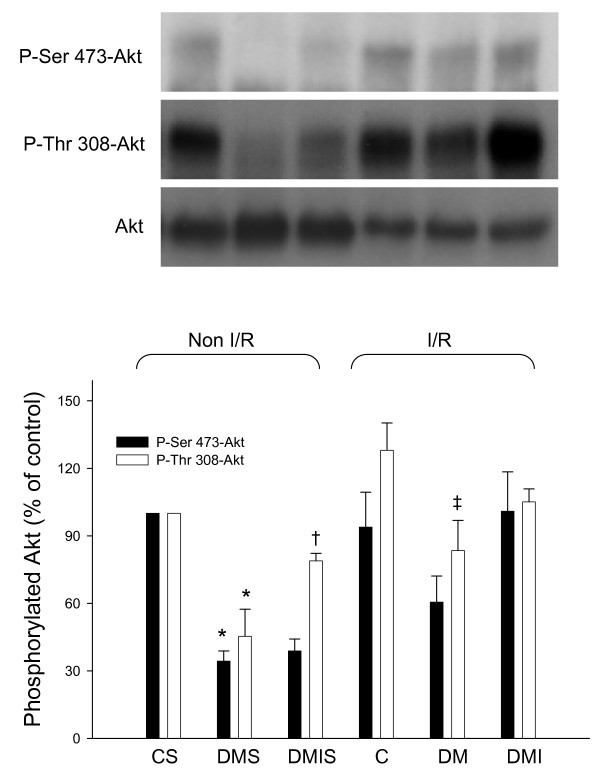
**The influences of diabetes on Akt and phosphorylated protein level were examined in the age-matched controlled, STZ-diabetic, and insulin-treated diabetic rats with or without I/R injury**. Equal amounts of proteins were resolved on 10% SDS-PAGE and blotted with respective Akt, p-ser 473-Akt, and p-thr 308-Akt antibodies. All experiments were performed in triplicates from six animals (*, vs. CS; †, vs. DMS; ‡, vs. C; n = 6 per group). Upper panels show blots, and quantified ratio is shown in the lower panels. C, age-matched control; CS, control sham; DM, STZ-induced diabetes; DMS, DM sham; DMI, diabetes treated with insulin (4 IU/day for 5 days); DMIS, DMI sham; I/R, myocardial ischemia 1 h following by a 3 h of reperfusion.

Under pathological conditions such as diabetes and myocardial ischemia, insulin signal transduction pathways, such as phosphatidylinositol 3-kinase/protein kinase B (Akt) signaling, are clearly modified [25,26]. Cardiac Akt protein expression and phosphorylation levels were examined in age-matched control, STZ-diabetic, and insulin-treated diabetic rats with and without I/R injuries. In the non-I/R heart, diabetes significantly reduced the level of phosphorylated Akt (P-Thr 308-Akt and P-Ser 473-Akt) protein compared to age-matched controls, while treatment of insulin yielded a higher phosphorylated Akt (P-Thr 308-Akt) level in comparison to the diabetic group (Figure 5). Under myocardial I/R injury, the capacity for Akt phosphorylation is impaired in insulin-deficient diabetes, and treatment of insulin failed to further induce the phosphorylation level of Akt (Figure 5).

## Discussion

The main findings of the study are as follows: (1) In STZ-induced hyperglycemic and hypoinsulinemic rats, the phosphorylated Akt and membranous GLUT4 protein levels were markedly reduced in the heart, suggesting that insulin-stimulated Akt/GLUT4 signaling was impaired in STZ-induced insulin-deficient diabetes. (2) The defect of insulin-stimulated Akt/GLUT4 signaling parallels with ventricular contractile dysfunctions, bradycardia, and hypotension in STZ-diabetic rats. These detrimental effects were partially reversed by 5 days of insulin treatment. (3) In animals subjected to acute myocardial I/R injury, a higher mortality rate was seen in the diabetic group and this phenomenon coincided with ventricular contractile dysfunctions, but not with arrhythmias or myocardial infarction since VT, VF, and necrotic area were not exacerbated in diabetic heart. (4) Acute myocardial I/R injury in diabetic rats markedly reduced cardiac output subsequent to bradycardia and reduction of contractility (including reduced end-systolic pressure, maximal slope of systolic pressure increment, and load-independent index of contractility). These alterations may be the leading causes of the high mortality rate in diabetic rats. (5) During the period of acute myocardial I/R injury, the diabetes-induced high mortality rate was almost completely prevented by insulin treatment. This beneficial effect of insulin may be related to the amelioration of I/R-induced arrhythmias and cardiac contractile function. On the other hand, the I/R-induced myocardial infarction (MI) was not attenuated by insulin treatment. Thus suggesting MI was not directly involved in the high mortality rate in diabetes.

Under physiological conditions, insulin regulates metabolism in the heart by modulating glucose transport, glycolysis, glycogen synthesis, lipid metabolism, protein synthesis, growth, contractility, and apoptosis [4,27,28]. In the present study, we used STZ (a most common islet cell toxin) to induce development of type 1 diabetes in SD rat. The metabolic features of STZ-diabetic rats include the prompt development of profound hyperglycemia (607 ± 16 mg/dl, Table 1)) and markedly reduced plasma insulin levels (1.08 ± 0.16 μg/L, Table 1). As such, the experimental animal model is particularly useful in examining the myocardial effects of hyperglycemia in the absence of hyperinsulinemia.

It is generally accepted that cardiac tissue obtains its energy via oxidation of various substrates like fatty acid (FA), glucose, lactate, and ketone bodies [29,30]. This flexibility in substrate selection is essential for the heart to maintain production of energy and contractile function. STZ-induced reduction in insulin would cause a compensatory shift in myocardial preference for fatty acids in the absence of insulin mediated glucose uptake. This leads to cardiac metabolism alterations and eventually impairment of contractile function [31,32]. Additionally, insulin-stimulated glucose uptake in cardiomyocytes is mediated primarily by the insulin responsive GLUT4. However, in addition to the basal cardiac glucose uptake mediated by GLUT1, contraction-mediated GLUT4 translocation to the sarcolemma may significantly contribute to myocardial glucose uptake [33]. As in other insulin-sensitive tissues, insulin signaling via PI3K/Akt pathways plays a key role in the regulation of GLUT4 translocation from cytoplasm to plasma membrane and subsequently in cardiac glucose uptake [34,35]. In the present study, the STZ-induced insulin deficient and hyperglycemic rat heart showed a reduction of Akt phosphorylation and GLUT4 translocation to plasma membrane as well as a decline in ventricular contractile functions. The diabetes-induced alterations on molecular (Akt/GLUT4) and organ levels (ventricular contractility) were seen in both I/R and non-I/R injured heart. Although treatment with insulin significantly re-established insulin-stimulated Akt/GLUT4 signaling and improved ventricular contractile function in basal (non-I/R) condition, insulin had a slight reverse effect in I/R injured diabetic rat heart. Whether or not the diabetes-decreased GLUT4 translocation to the plasma membrane was simply a result from insulin signaling deficiency or was an impairment of contraction-mediated GLUT4 translocation involved still needs to be clarified since diabetes also reduced ventricular contractility.

It has been shown that acidosis produced by myocardial ischemia provokes the inhibition of the tyrosine kinase activity of insulin receptor. This inhibition directly correlates to the decrease in phosphorylation of different elements downstream including PKB/Akt, p70S6K, and GSK-3 [36]. Even if the insulin signaling pathways were blunted during ischemia, insulin would still be able to act during reperfusion. Our result shows that myocardial I/R markedly increased GLUT4 translocation to plasma membrane, but had no effect on Akt phosphorylation in control animals without diabetes. This data may provide indirect evidence to suggest that glucose uptake was increased in I/R injured heart since membranous GLUT4 levels was elevated. Increasing glucose uptake may possibly shift myocardial metabolism from long chain fatty acid (LCFA) to glucose oxidation during reperfusion, which is more oxygen efficient and prevents the production of toxic LCFA intermediates. As seen from the rat models, diabetes seemed to blunt those compensatory effects. This may be the explanation for the low contractility and high mortality found in diabetic rats with I/R injury.

The in-vivo animal and human studies have clearly shown that diabetes and insulin resistance aggravate myocardial ischemic injury. However, many experimental studies using animal models of diabetes show widely varied results ranging from no change, increased, or decreased sensitivity of ischemia with or without reperfusion injury [37-41]. Our study provided systemic in-vivo evidence in different structural levels to explain how diabetes can aggravate myocardial I/R injury and revealed the role of insulin signaling. Our results show that diabetes with I/R injury reduced Akt/GLUT4 signaling (molecule level) without exacerbating myocardial infarction (tissue level) and arrhythmias (organ level), attenuated ventricular contractility (organ level), and raised mortality rate (organism system).

## Abbreviations

BP: blood pressure; CAD: coronary artery disease; CO: cardiac output; CVD: cardiovascular diseases; Ea: arterial elastance; ECG: electrocardiogram; EDP: end-diastolic pressure; EDPVR: end-diastolic PV relation; EDV: end-diastolic volume; Ees: end-systolic elastance; EF: ejection fraction; ESP: end-systolic pressure; ESPVR: end-systolic PV relation; ESV: end-systolic volume; FA: fatty acid; GLUT: glucose transporter; HR: heart rate; IR: insulin receptor; I/R: ischemia-reperfusion; LV: left ventricle; MAP: mean arterial pressure; max dP/dt: maximal slope of systolic pressure increment; min dP/dt: diastolic decrement; MI: myocardial infarction; PI3K: phosphatidylinositol 3-kinase; SV: stroke volume; SW: stroke work; TPR: total peripheral resistance; VF: ventricular fibrillation; VT: ventricular tachycardia; STZ: streptozotocin.

## Competing interests

The authors declare that they have no competing interests.

## Authors' contributions

JPH, SSH, and LMH conceived the hypothesis, contributed to the design and conduct of the study, conducted the statistical analyses, drafted the manuscript and critically revised manuscript. JYD provided important comments and excellent techniques in the paper. All authors read and approved the final manuscript.
